# N-Doped Carbon-Coated ZnS with Sulfur-Vacancy Defect for Enhanced Photocatalytic Activity in the Visible Light Region

**DOI:** 10.3390/nano9121657

**Published:** 2019-11-21

**Authors:** Hao Peng, Daixin Liu, Xiaogang Zheng, Xiaojin Fu

**Affiliations:** 1 College of Chemistry and Chemical Engineering, Yangtze Normal University, Chongqing 408100, China; 2 College of Chemistry and Chemical Engineering, Neijiang Normal University, Neijiang 641100, China; ldx18181976958@163.com (D.L.); fu-xj2007@163.com (X.F.)

**Keywords:** ZnS, sulfur-vacancy defect, N-doped carbon, visible-light harvesting

## Abstract

In this work, N-doped carbon-coated ZnS with a sulfur-vacancy defect (ZnS@N-C) was performed for the visible-light-driven photodegradation of tetracycline hydrochloride (TCH). The obtained ZnS@N-C exhibited enhanced photocatalytic activity compared with ZnS for TCH removal. Among these ZnS@N-C composites, ZnS@N-C-3 with N-doped content of 3.01% (100 nm) presented the best visible-light photocatalytic activity and superior long-term photocatalytic stability after five cycle times for TCH removal in the visible light region. This may be ascribed to the interface between the N-doped carbon shell and ZnS with a sulfur-vacancy defect for efficient charge transfer and the restrained recombination of charge carriers. Electron spin resonance (ESR) results indicate that the ·O_2_^‒^ radical plays a crucial role in the enhanced photocatalytic activity of ZnS@N-C-3.

## 1. Introduction

Photocatalysis, as a potential route to relieving environmental and energy issues, has been intensively applied for pollutant degradation [1,2,3,4], water splitting [5,6,7], and solar energy conversion [8,9,10,11]. As a typical metal sulfide, ZnS, which has a large band gap (3.6~3.8 eV), exhibits excellent photocatalytic capacity, owing to its strong oxidation and high negative potentials of excited electrons [12,13]. However, the photocatalytic activity of ZnS is greatly affected by its structural and optical properties, especially defects such as sulfur vacancy, sulfur interstitial, zinc vacancy, and zinc interstitial. This may be ascribed to the crucial role of the lattice defects, exposed facets, and crystalline phases in dominating light-harvesting capacity, regulating active sites or facets, and managing charge transfer kinetics [14,15,16]. Sulfur vacancies are introduced by allowing the orbital of the neighboring zinc atoms to relax. Sulfur vacancies not only induce the raising of the valence band position but also serve as photosensitization units and hole acceptors, enhancing the visible light response, charge carrier separation, and resistance to photo-corrosion [17,18]. Hence, it is crucial to design and explore ZnS-based heterojunctions with efficient visible light sorption and affluent vacancy sites.

Although the sulfur defects of ZnS can narrow the band gap and act as sorption sites for fascinating charge transfers and restraining electron-hole combinations, excessive amount defects act as recombination centers for inferior photocatalytic activity. Recently, carbon materials such as carbon dots and graphene have been widely confirmed as effective photosensitizers in catalytic ozonation and photocatalytic processes due to their low cytotoxicity and high mobility of photo-produced electrons [19,20,21,22]. The conjugated π structure of carbon is suitable for the enhanced interactivity of composites between carbon and semiconductors. To further enhance the electron transfer and extend the visible light harvesting region, carbon doped with N atoms can adjust the work function of carbon and induce charge delocalization [23,24,25,26]. Hsu et al. have reported that sandwich-like hydrogenated C-doped anatase TiO_2_ nanocrystals/N-doped carbon dots@layer/rutile TiO_2_ nanorod arrays have exhibited efficient photoelectron-chemical activity of water oxidation [27]. This may be attributed to the N-doped carbon dots being the photosensitizer of long wavelength light-harvesting and the N-doped carbon layer being the conductive layer of charge transport toward the current collector. Wang et al. have also confirmed that N-doped carbon dots/g-C_3_N_4_ can enhance visible-light photo-degradation activity for indomethacin due to the efficient charge separation and band gap narrowing of N-doped carbon dots [28]. Core-shell structured ZnO@N-doped carbon can efficiently adsorb and photodegrade methylene blue in the visible light region [29]. Electrons generated from nitrogen are transferred from the N-doped carbon surface to adsorbed oxygen, forming reactive radicals in the photocatalytic system [30,31,32,33,34]. In addition, surface polarity of N-doped carbon can promote dispersion in aqueous solution. However, few works have focused on the enhanced photocatalytic activity of ZnS with sulfur-vacancy defects using N-doped carbon coating.

This work focuses on the synthesis of ZnS with an N-doped carbon coating (ZnS@N-C) and its sulfur-vacancy effect on visible-light photocatalytic activity for the removal of tetracycline hydrochloride (TCH). Reported photocatalysts such as CuInS_2_/Mg(OH)_2_ [35], ZnIn_2_S_4_/BiPO_4_ [36], AgBr/Bi_2_WO_6_ [37], BiOI/g-C_3_N_4_/CeO_2_ [38], and TiO_2_/BiOCl [39] are efficient heterojunctions for the visible-light-driven degradation of TCH. Differently from these compositions, N-doped carbon serves as the electron capture, facilitating the separation of electron-hole pairs. In this strategy, N-doped carbon is generated from the decomposition of cyanamide and coated on the surface of ZnS nano-particles, which are further treated in N_2_ to obtain a sulfur vacancy. The sulfur-vacancy defect and N-doped carbon of ZnS@N-C is designed to enhance the charge transfer and electron-hole separation and extend the light sorption toward the visible light region.

## 2. Materials and Methods

### 2.1. Preparation of Catalysts

ZnS nanoparticles were prepared via a hydrothermal route. Briefly, 1 mmol zinc nitrate (Zn(NO_3_)_2_·6H_2_O, AR), 1 mmol thiourea (CH_4_N_2_S, AR), and 2 g polyvinyl pyrrolidone (PVP, M = 58,000) were added to 150 mL acetonitrile (C_2_H_3_N, AR) with vigorous stirring at room temperature for 2 h, and then transferred to a Teflon-lined autoclave (200 mL) with hydrothermal treatment at 473 K for 5 h. When cooled to room temperature, the above product was filtered, washed with anhydrous ethanol three times, and dried at 333 K for 10 h.

ZnS@N-C nanoparticles were synthesized by a hydrothermal route. In a typical process, 0.1 g obtained ZnS bulks, 600 μL cyanamide (CH_2_N_2_, AR), and 1.5 g PVP were dispersed in an 80 mL ethanol solution consisting of anhydrous ethanol (40 mL) and deionized water (40 mL), and then stirred at room temperature for 2 h. The above solution was sealed in a Teflon-lined autoclave (100 mL) at 433 K for 7 h. After cooling to room temperature, the suspension was centrifuged, washed with anhydrous ethanol and deionized water, dried at 323 K for 10 h, and calcined at 773 K for 2 h under an N_2_ atmosphere (40 mL min^−1^) to obtain ZnS@N-C (ZnS@N-C-1). ZnS-N-C-X (X=2, 3, 4, and 5) with varying N-doped C content was prepared using varying cyanamide content (600, 700, 800, and 1000 μL) according to the above process.

### 2.2. Characterization of Catalysts

ZnS@N-C composites were characterized by X-ray powder diffraction (Bruker D8, Karlsruhe, Germany) with Cu Kα radiation (λ = 1.54 Å) at 2θ ranging from 20° to 80° and a scan rate of 4.0° min^−1^, scanning electron microscopy (Hitachi S-4800, Tokyo, Japan) at an acceleration voltage of 5 kV, transmission electron microscopy (TEM, JEM-2010, Tokyo, Japan) atan accelerating voltage of 200 kV, high resolution transmission electron microcopy (HRTEM, JEOL 4000EX, Tokyo, Japan) at an accelerating voltage of 200 kV, N_2_ adsorption-desorption (NOVA 2200e, Boynton Beach, FL, USA) using adsorption of N_2_ at 77 K, Fourier transform infrared spectroscopy (Bruker VECTORTM 22, Karlsruhe, Germany) equipped with a diamond-attenuated total reflectance (ATR) cell (Specac Golden Gate MkII, Karlsruhe, Germany) and spectral range 600–4000 cm^−1^, X-ray photoelectron spectroscopy (XPS, ESCALAB 250, Waltham, MA, USA) with a monochromatic Al Kα X−ray source (1486.6 eV) under high vacuum of 2 ×10^−7^ Pa, UV-Vis diffuse reflectance spectroscopy (UV-Vis DRS, Hitachi U-4100, Tokyo, Japan) with BaSO_4_ serving as the reflectance standard and an integrating sphere attachment within a range of 200–900 nm, photoluminescence (PL, FLSP 920, Edinburgh, UK)using an excitation wavelength of 325 nm at room temperature, and electronspin resonance (ESR, JES-FA200, Tokyo, Japan) with 5,5-dimethyl-1-pyrroline N-oxide (DMPO) serving as the capture agents of photo excited radicals.

### 2.3. Photocatalytic Activity

ZnS@N-C composites were applied for the visible-light-driven degradation of TCH using a Xe lamp irradiation (300 W). Briefly, 100 mg obtained bulks were dispersed into a 100 mL TCH solution (50 mg L^−1^) and stirred in a dark room for 2 h to reach an adsorption-desorption equilibrium. After certain time intervals, a 5 mL solution was sampled and analyzed by UV-visible absorption spectra (UV-4802, Unico Instrument Co., Ltd., Shanghai, China). The photocatalytic stability of ZnS@N-C was carried out for five cycle times observed according to the above experimental process. The used ZnS@N-C bulks after each cycle testing were separated, washed three times with deionized water, and again dispersed in TCH solution for the next cycle. Quenching reagents such as tert-butyl alcohol (*t*-BuOH), p-benzoquinone (*p*-BQ), and ethylenediamine tetraacetic acid disodium salt (EDTA-2Na) were performed for the evaluation of radical species such as ·OH, ·O_2_^‒^, and h^+^ in photocatalytic reactions according to the above process.

## 3. Results

**Figure 1:**
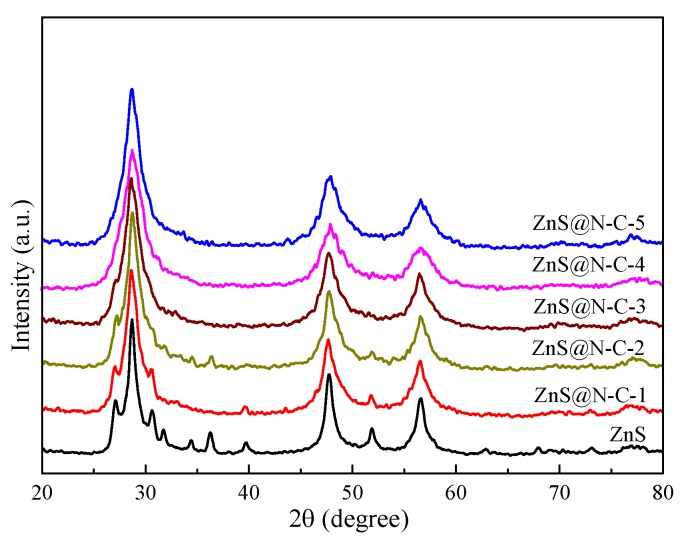
XRD patterns of ZnS and N-doped carbon-coated ZnS with a sulfur-vacancy defect (ZnS@N-C) composites.

The X-ray powder diffraction (XRD) patterns of ZnS and ZnS@N-C with varying N-doped carbon content are depicted in Figure 1. The peaks at 27.2°, 28.6°, 30.6°, 47.8°, 51.9°, and 56.7° in the XRD patterns of ZnS, ZnS@N-C-1, and ZnS@N-C-2 are respectively indexed as the (100), (002), (101), (110), (103), and (112) facets of wurtzite hexagonal ZnS (JCPDS, 36-1450) [18,30]. The wurtzite and sphalerite (cubic) phases of ZnS are formed at a high temperature above 1293 K and at room temperature, respectively [40]. Cubic ZnS can be transformed into hexagonal ZnS in a hydrothermal system at around 500 K. During the sintering process, the precursor of N-doped carbon generated from cyanamide is graphitized and decomposed into H_2_O and NO_x_ molecules that further react with ZnS particles, leading to a phase transform and the crystal growth of ZnS. Wurtzite particles only grow on the surface of coarsened sphalerite particles, and the phase stability of nanoscale ZnS is intensively affected by particle size [41]. With increasing N-doped carbon content, the peaks of ZnS@N-C-X (X = 3, 4, and 5) at 28.6°, 47.8°, and 56.7° are respectively indexed as the (111), (220), and (311) planes of sphalerite ZnS (JCPDS, 05-0566) [18].

**Figure 2:**
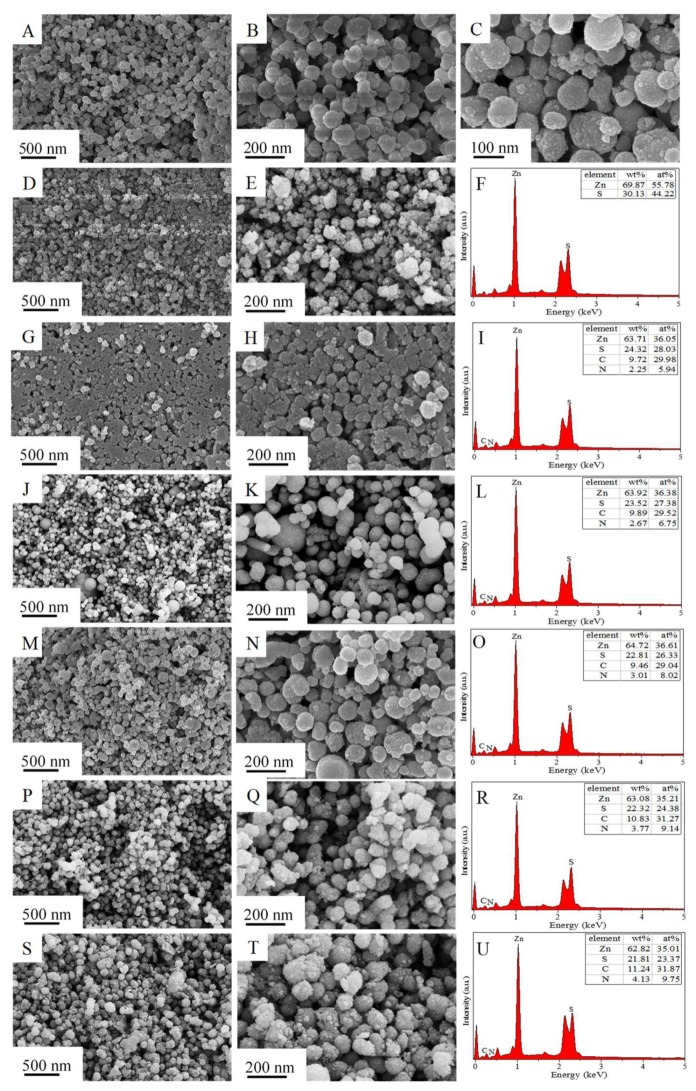
Scanning electron microscopy (SEM) images and the corresponding energy dispersive X-ray analysis(EDX) patterns of ZnS (**A**–**C**), calcined ZnS (**D**–**F**), ZnS@N-C-1 (**G**–**I**), ZnS@N-C-2 (**J**–**L**), ZnS@N-C-3 (**M**–**O**), ZnS@N-C-4 (**P**–**R**), and ZnS@N-C-5 (**S**–**U**).

**Figure 3:**
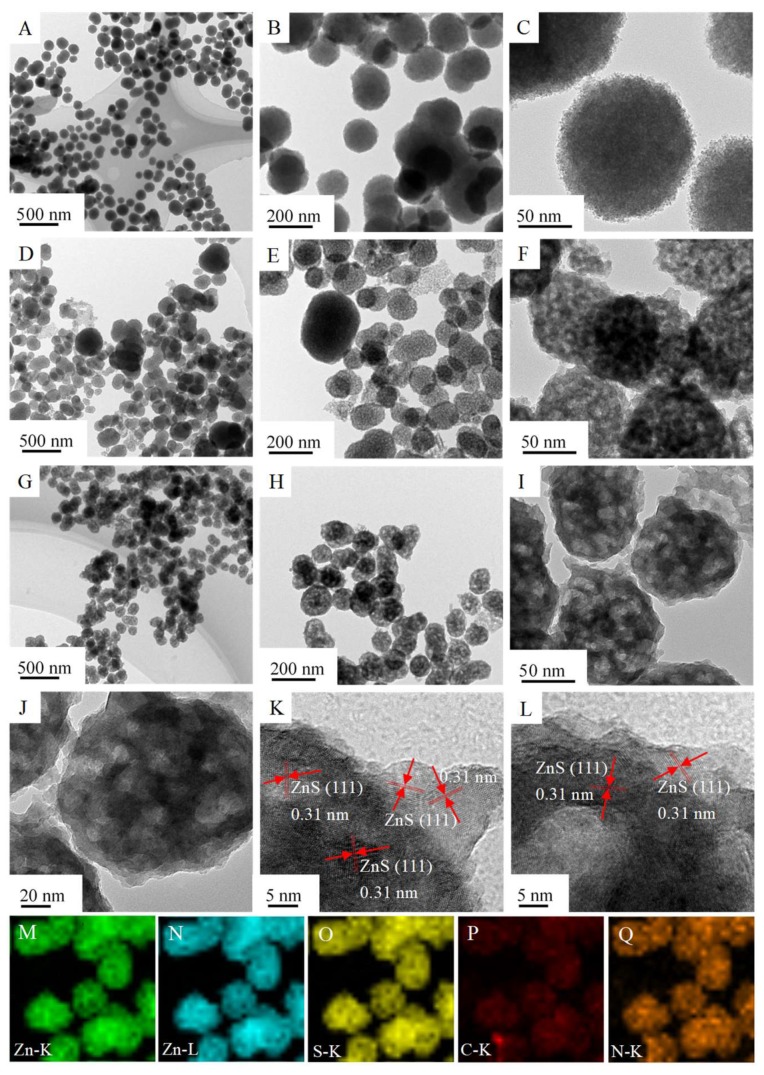
Transmission electron microscopy (TEM) images of ZnS (**A**–**C**), calcined ZnS (**D**–**F**) and ZnS@N-C-3 (**G**–**J**); high resolution TEM (HRTEM) images (**K**,**L**) and element mapping images (**M**–**Q**) of ZnS@N-C-3.

ZnS particles are dispersive and amorphous nanospheres (average diameter 100 nm) which consist of ultrafine ZnS nanoparticles (<10 nm), as shown in Figure 2A–C and Figure 3A–C. After being calcined at 773 K, part of the ZnS nanospheres was disintegrated into smaller particles (<100 nm) and the other part was agglomerated into larger particles (>150 nm) with an amorphous and porous structure (Figure 2D,E and Figure 3D–F). In contrast with calcined ZnS, N-doped carbon can efficiently restrain the disassembly and agglomeration of ZnS, especially with low content of N-doped carbon (Figure 2G,H). With a further increase in N-doped carbon content, the morphology structures of obtained ZnS@N-C composites are not seriously affected due to the shielding effect of N-doped carbon, as shown in Figure 2J–T. The N-doping content, N-doping atomic ratio, and carbon content of ZnS@N-C composites increases with an increase in the cyanamide volume. The Zn/S atomic ratios of ZnS and ZnS@N-C are higher than those predicted by theoretical values, indicating the presence of sulfur-vacancy defects. ZnS@N-C-3 is a porous and irregular sphere with an average size of 100 nm (Figure 3G–J). The (111) facet of cubic ZnS with a spacing distance of 0.31 nm was detected in ZnS@N-C-3 (Figure 3K,L), which is in agreement with its XRD pattern [30]. Energy dispersive X-ray (EDX) results (Figure 2F,I,L,O,R,U) suggest that the Zn and S elements exist in calcined ZnS, and the N and C elements are also detected in ZnS@N-C. Elemental mapping images (Figure 3M–Q) also confirm the existence of Zn, S, C, and N elements in ZnS@N-C-3, indicating the formation of an N-doped carbon coating.

**Figure 4:**
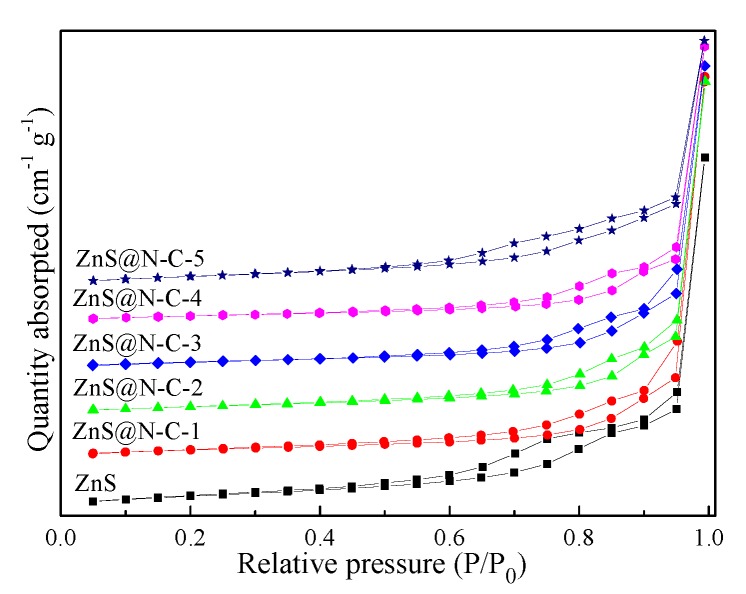
N_2_ adsorption–desorption isotherms of ZnS and ZnS@N-C composites.

**Figure 5:**
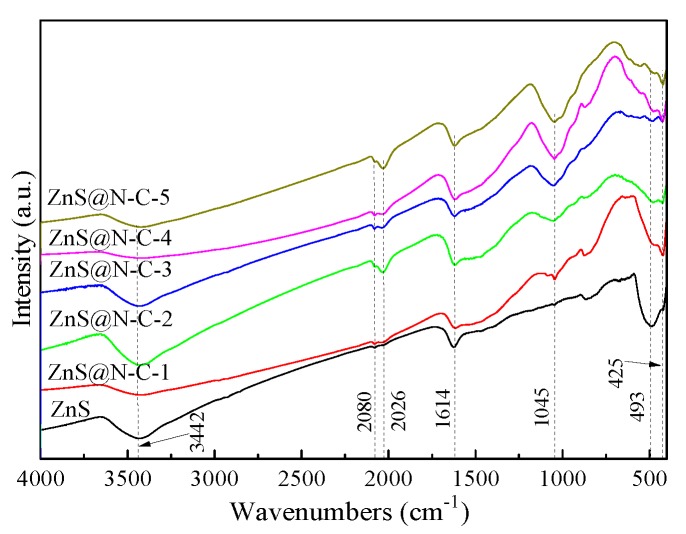
Fourier Transform infrared spectroscopy (FT-IR) spectra of ZnS and ZnS@N-C composites.

N_2_ adsorption–desorption isotherms of ZnS and ZnS@N-C samples (Figure 4) are typical IV isotherms, forming irregular mesopores and/or macropores. The specific surface area of ZnS@N-C increases and then decreases with an increase in N-doped carbon content. Although increasing N-doped carbon content is favorable for the reduced probability of a destroyed texture structure, an N-doped carbon coating takes up the pore structure of ZnS and even results in aggregation at a high sintered temperature, as listed in Table 1. The peaks of ZnS and ZnS@N-C at around 3442 cm^−1^ and 1614 cm^−1^ (Figure 5) can be attributed to the stretching vibration and bending vibration of an O-H group [42,43,44,45,46]. The peaks of ZnS@N-C composites at 2080 cm^−1^ and 2026 cm^−1^ can be assigned to the stretching vibration of C≡C and C=N groups, respectively. The peak at 1045 cm^−1^ can be assigned to the C–N band [44]. The peaks at 493 cm^−1^ and 425 cm^−1^ can be ascribed to the stretching vibration of the Zn–S band [47].

**Figure 6:**
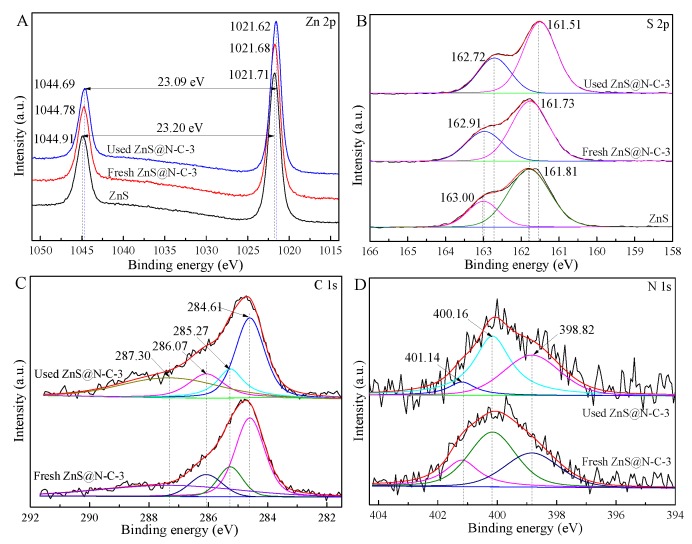
Zn 2p (**A**), S 2p (**B**), C 1s (**C**), and N 1s (**D**) XPS spectra of ZnS and fresh and used ZnS@N-C-3.

**Figure 7:**
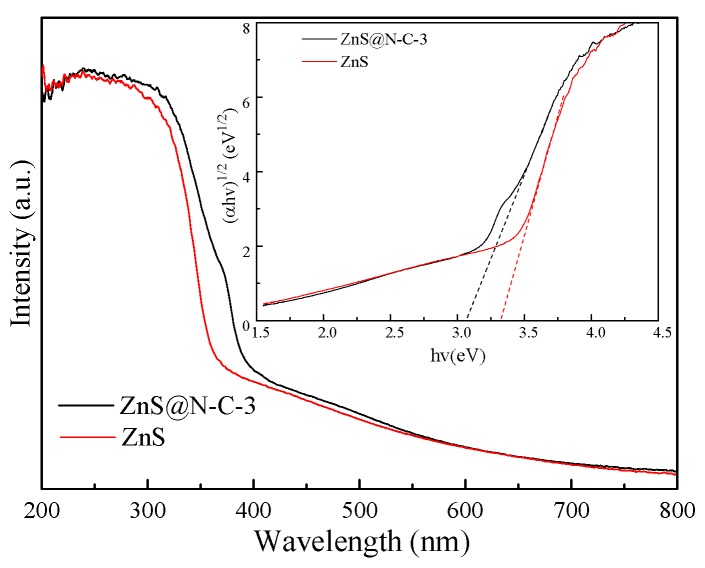
UV–vis diffuse reflectance spectroscopy (DRS) spectra and band gap analysis plot of ZnS and ZnS@N-C-3.

As shown in Figure 6A, the binding energy peaks of Zn 2p_1/2_ and Zn 2p_1/2_are located, respectively, at 1044.91 eV and 1021.71 eV in the Zn 2p XPS spectrum. These Zn 2p_1/2_ and Zn 2p_3/2_ peaks of fresh ZnS@N-C-3 (1044.78 eV and 1021.68 eV) and a used sample (1044.69 eV and 1021.62 eV) are shifted to lower binding energies in comparison with ZnS. The former may be ascribed to the capture-effect of the N-doped carbon coating and the latter may be attributed to photo-corrosion which occurs after five cycles. The separation of Zn 2p_1/2_ and Zn 2p_3/2_ peaks in fresh and used ZnS@N-C-3 are, respectively, 23.20 eV and 23.09 eV, leading to the reduced electron density of Zn 2p. In contrast with S 2p (163.00 eV and 161.81 eV), the binding energy peaks of S 2p_1/2_ and S 2p_1/2_ of fresh (163.91 eV and 161.73 eV) and used (162.72 eV and 161.51 eV) ZnS@N-C-3 samples appear at a lower binding energy, as shown in Figure 6B. It may be noted that the Zn/S atomic ratios of fresh ZnS@N-C-3 (1.80), ZnS (1.67), and used ZnS@N-C-3 (1.73) are higher than that predicted by the theoretical value (1.0) of ZnS, which further indicates the presence of sulfur-vacancy defects in the obtained ZnS-based samples. The C 1s spectra of fresh and used ZnS@N-C-3 (Figure 6C) are divided into four Gaussian curves, of which the typical peaks at 287.30 eV, 286.07 eV, 285.27 eV, and 529.1 eV may be assigned, respectively, to C–C, C–O/C–N, C=O/C=N, and COO– groups. The varying area ratio of these binding energy peaks leads to differences in the C 1s XPS spectra of fresh and used ZnS@N-C-3 [29,34,48]. The split peaks of fresh and used ZnS@N-C-3 (Figure 6D) at 401.14 eV, 400.16 eV, and 398.82 eV may be attributed to C–N, C–O, and C=O, respectively. The area ratio of these binding energy peaks is responsible for the varying N 1s XPS spectra [49,50]. Compared with ZnS, ZnS@N-C-3 exhibits an enhanced visible-light sorption capacity (Figure 7). Based on the plot of (αhv)^1/2^ versus (hv), the calculated band gap energies of ZnS and ZnS@N-C-3 are, respectively, 3.32 eV and 3.06 eV. Due to the formation of a new mid-gap state, the conduction band (CB) of obtained ZnS with abundant sulfur vacancies is lower than that of ZnS, leading to the band gap energy of obtained ZnS being lower than that in previous works (3.2~3.6 eV) [51,52,53,54,55]. N-doped carbon can modulate the content of sulfur vacancies within the ZnS nanocrystals, leading to a reduced band gap energy of ZnS@N-C-3.

**Figure 8:**
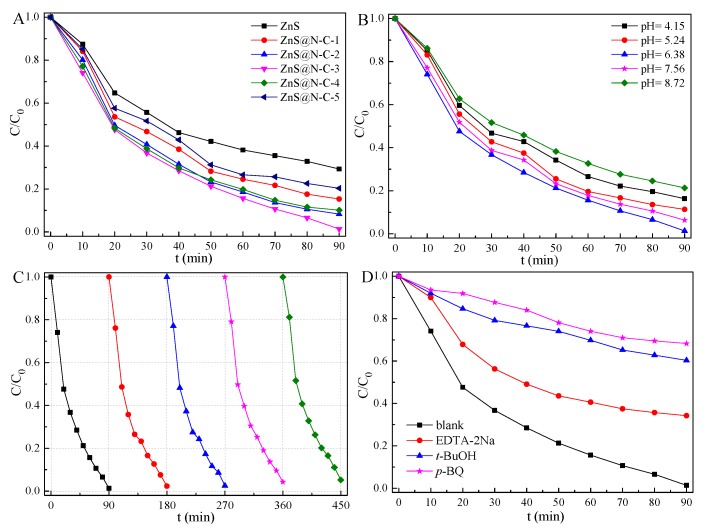
Photocatalytic activity of ZnS and ZnS@N-C composites (**A**), effect of pH value on the photocatalytic capacity of ZnS@N-C-3 (**B**), photocatalytic durability of ZnS@N-C-3 (**C**)for five cycles, and quenching testing of ZnS@N-C-3 (**D**) for tetracycline hydrochloride (TCH) removal.

The obtained samples were performed for visible-light-driven photocatalytic activity for TCH removal. Although the specific surface area and pore volume are important for sorption capacity, the surface-functionalization of N-doped carbon is much more significant for its sorption capacity and photocatalytic performance. In contrast with ZnS, ZnS with an N-doped carbon coating exhibits improved photocatalytic activity (Figure 8A). It has been indicated that N-doped carbon and sulfur vacancies are likely to promote the separation of charge carriers, leading to boosted quantum efficiency [49,51]. The photocatalytic capacity of ZnS@N-C increases and then decreases with an increase in N-doped carbon content (Figure 8A) as well as pH (Figure 8B). In such a photocatalytic system, H_2_O molecules can diffuse from the TCH solution into the interface of the N-doped carbon and ZnS and then react with h^+^ radicals to generate OH groups [28]. The dissolved O_2_ molecules can react with photoexcited electrons (e^−^) to generate ·O_2_^−^ radicals, which further react with H^+^ ions to form ·OOH and H_2_O_2_ species under Lewis acid conditions. H_2_O_2_ molecules, with the assistance of e^−^, are converted into ·OH radicals and OH^−^ ions. It may be noted that OH^−^ ions are intensively consumed by h^+^ to produce ·OH radicals under Lewis base conditions. An optimized pH value is suitable for the above photocatalytic process. Hence, ZnS@N-C-3 with N-doped content of 3.01% exhibits the best visible-light photocatalytic activity for TCH removal at a pH value of 6.38. The obtained ZnS@N-C-3 (Table 2) exhibits higher photocatalytic activity than that in some reported works. The visible-light-driven long-term durability of ZnS@N-C-3 (Figure 8C) is slightly affected after five cycles due to photo-corrosion in the photocatalytic system, which is confirmed by the XPS spectrum of ZnS@N-C-3 (Figure 6). Similarly, the photocatalytic activity of TiO_2_ is greatly affected by its crystalline phases, microstructure, and naked lattice planes [56,57,58,59,60]. ZnS@N-C-3, as well as TiO_2_-based composites, can be considered an efficient photocatalyst for the visible-light-driven photodegradation of textile and antibiotic effluents in industrial application.

**Figure 9:**
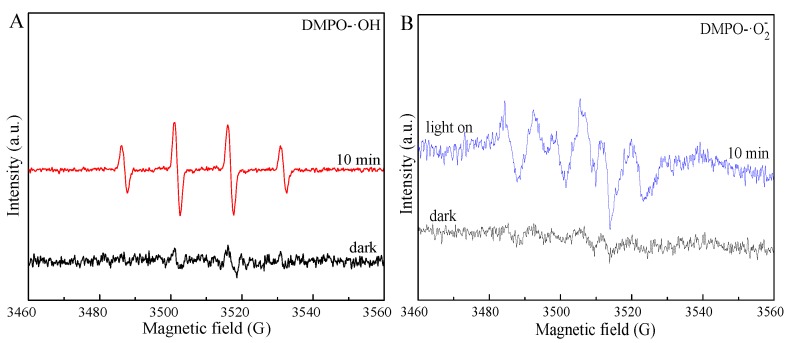
Electron spin resonance (ESR) spectra of ZnS@N-C-3 for 5,5-dimethyl-1-pyrroline N-oxide(DMPO)-·OH in aqueous solution(**A**) and DMPO-·O_2_^−^ in methanol (**B**).

**Figure 10:**
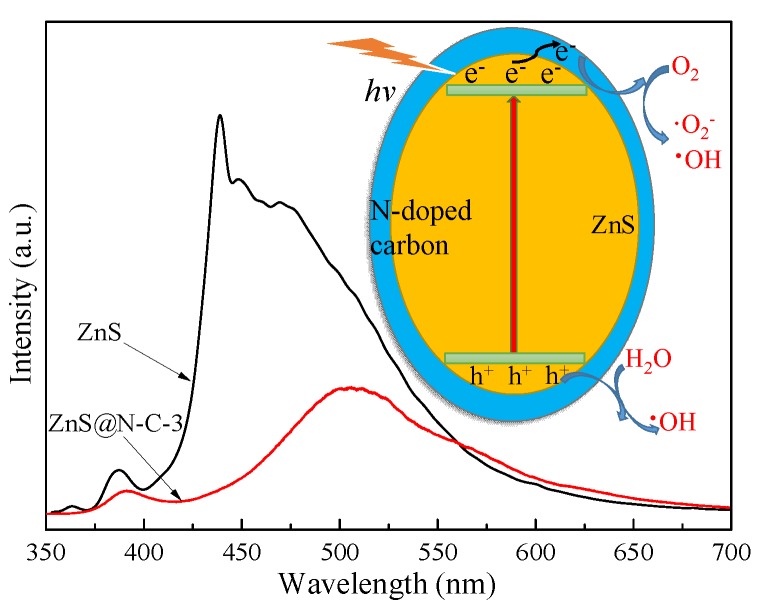
Photoluminescence (PL) spectrum and photocatalytic mechanism of ZnS@N-C-3.

The photocatalytic capacity of ZnS@N-C-3 (Figure 8D) is greatly restrained by the presence of *p*-BQ and *t*-BuOH. It has been indicated that ·O_2_^−^ and ·OH species are the main radicals of ZnS@N-C-3 for TCH removal. After being irradiated for 10 min, the ESR intensity of DMPO-·O_2_^−^ (Figure 9) is stronger than that of DMPO-·OH, highlighting the crucial role of ·O_2_^−^ radicals in the enhanced photocatalytic activity of ZnS@N-C-3. ZnS@N-C-3 (Figure 10) exhibits weaker PL intensity compared with ZnS, indicating that the suppressed recombination rate of electron-hole pairs and the efficient separation of charge carriers are responsible for the enhanced photocatalytic activity [42,43,47]. A possible photocatalytic mechanism of ZnS@N-C-3 is that the photo-excited electrons escape from the valence band (VB) of ZnS and then are transferred to N-doped carbon through the interface of ZnS with abundant sulfur-vacancy defects and N-doped carbon, causing efficient charge transfer and separation and leaving holes for the generation of reactive species [51,53]. The trapped electrons can transfer to the surface of the N-doped carbon coating and then react with O_2_ molecules to yield ·OH and ·O_2_^−^species [55]. The hole radicals located in the CB of ZnS can react with H_2_O molecules to yield ·OH radicals. With the assistance of the above radicals in the visible light region, TCH moleculesare decomposed via the N–C bond cleavage and hydroxylation and further react with ·OH radicals to destroy the C2-C3 double-bond and eliminate NH_3_, and subsequently are disintegrated into small molecules and even H_2_O and CO_2_ [35].

## 4. Conclusions

In this work, ZnS coated with N-doped carbon exhibited improved photocatalytic activity for TCH removal in the visible light region compared with ZnS. Among these composites, ZnS@N-C-3 with N-doped content of 3.01% exhibited the best visible-light photocatalytic activity for TCH removal at the pH value of 6.38. Its long-term durability was slightly affected after five cycles. The synergistic effect between N-doped carbon and ZnS with sulfur-vacancy defects is responsible for the enhanced photocatalytic activity and stability. Efficient charge transfer and restrained electron-hole recombination are obtained at the interface of the N-C coating and ZnS core. N-doped carbon is a promising strategy to enhance the photocatalytic capacity of semiconductors.

## Figures and Tables

**Table 1:** Surface parameters of ZnS and ZnS@N-C samples.

Samples	Surface Area (m^2^ g^−1^)	Pore Volume (cm^3^ g^−1^)	Pore Diameter (nm)
ZnS	72.82	0.58	6.58
ZnS@N-C-1	39.95	0.47	9.95
ZnS@N-C-2	42.83	0.54	9.67
ZnS@N-C-3	46.46	0.61	9.55
ZnS@N-C-4	45.57	0.58	9.61
ZnS@N-C-5	43.58	0.49	9.56

**Table 2:** Comparison of the degradation rate of various materials towards pollutants.

Material	Dosage (mg)	Light Source	Pollutant	Time (min)	Degradation Efficiency (%)	Ref.
α-Fe_2_O_3_@TiO_2_	100	Xe (300 W)	Tetracycline (50 mg L^−1^, 200 mL)	90	100	[4]
MgO@N-C	100	Xe (300 W)	Methylene blue (120 mg L^−1^, 100 mL)	70	98.67	[22]
N-C/g-C_3_N_4_	50	Xe (350 W)	Indomethacin (4 mg L^−1^, 50 mL)	90	91.75	[28]
ZnS@N/S-C	20	Xe (300 W)	Bisphenol-A (200 mg L^−1^, 50 mL)	200	88.00	[31]
ZnS@N-C	50	Hg (150 W)	Methylene blue (10 mg L^−1^, 50 mL)	110	97.20	[32]
Sm_2_O_3_@N-C	100	Xe (300 W)	Tetracycline (50 mg L^−1^, 100 mL)	120	90.26	[34]
ZnS-15%RGO	20	Xe (300 W)	Methylene blue (20 mg L^−1^, 100 mL)	240	89.43	[55]
ZnS@N-C-3	100	Xe (300 W)	Tetracycline (40 mg L^−1^, 100 mL)	90	98.60	This work
